# Prevalence of Geographic Tongue and Related Predisposing Factors in 7-18 Year-Old Students in Kermanshah, Iran 2014

**DOI:** 10.5539/gjhs.v7n5p91

**Published:** 2015-02-24

**Authors:** Fatemeh Rezaei, Mina Safarzadeh, Hamidreza Mozafari, Payam Tavakoli

**Affiliations:** 1 Oral Medicine Department, School of Dentistry, Kermanshah University of Medical Sciences, Kermanshah, Iran; 2 School of Dentistry, Kermanshah University of Medical Sciences, Kermanshah, Iran; 3 Pathology Department, School of Dentistry, Kermanshah University of Medical Sciences, Kermanshah, Iran

**Keywords:** benign migratory glossitis, fissured tongue, geographic tongue, psoriasis

## Abstract

Geographic tongue is a benign lesion at the dorsum and margins of the tongue that sometimes causes pain and burning sensation. This lesion is characterized by an erythematous area with white or yellow folded edges. The predisposing factors of this lesion include heredity, allergies, psoriasis, stress, fissured tongue and consumption of some foods. The present study was conducted to investigate the prevalence of geographic tongue and its related factors among the 7-18 year-old students in Kermanshah, Iran. This descriptive cross-sectional study was carried out in three schools in Kermanshah using multi-stage random cluster sampling method. A total number of 3600 students were examined (1800 girls and 1800 boys). Demographic data and the results of examinations were recorded in a questionnaire. The factors affecting the incidence of geographic tongue were analyzed by the SPSS-20 software and the Chi-square test. The prevalence of geographic tongue was 7.86% (283 individuals). The incidence of this lesion was significantly higher in males than in females (p<0.01). There was no relationship between geographic tongue and psoriasis or fissured tongue. Pain and discomfort during eating was more prevalent in those with geographic tounge compared to those without this condition (p<0.02). The prevalence of geographic tongue among the studied population was 7.86%, and the prevalence of geographic tongue in male students was higher than in female students.

## 1. Introduction

Geographic tongue or benign migratory glossitis is a benign and common inflammatory phenomenon. Atrophy of the filiform papillae leaves an erythematous area with a white, yellow or slighty gray elevated peripheral zone, and irregular jagged pattern (like a geographic map) of the tongue. This condition usually involves the dorsal surface and lateral borders of the tongue (Greenburg & Glick, 2014; Shafer et al., 2010; Ishibashi et al., 2010).

The prevalence of this lesion has been reported to be 2% among the population in the United States (Greenburg et al., 2014). This lesion has been observed in various ages, and no definite correlation has been reported between the age and gender distribution and fequency of this lesion (Richardson, 1968). The geographic tongue lesion is usually asymptomatic (Greenburg & Glick, 2014; Ugar-Cankal et al., 2005) and the patient is often unaware of its existence. However, in some cases the lesion reacts to hot and salty or spicy foods as well as alcoholic drinks in adults (Scully, 2004; Regezi et al., 2003). Also, worsening of the lesion due to dental treatments has been reported in children (Prinz, 1927). It is characterised by exacerbation and remission periods and the severity of symptoms depends on the disease activity (Cawson et al., 2001). During the severe symptomatic stage, lesions may be accompanied by mouth irritation, burning, and sensing a foreign body or pain in the ears or submandibular lymph nodes on the same side (Waltimo, 1991 Salem et al., 1987; Sigal & Mock, 1992). In spite of the presence of some reports about the factors involved in etiology of geographic tongue, its mechanism has not definitely been determined.

But it seems that patients with a family history and allergy-like asthma (Barton et al., 1982), eczematic dermatitis (Regezi et al., 2003) and hay fever or in general those with a high level of serum immunoglobulin E are more prone to be afflicted with geographic tongue than other people without these characteristics (Greenburg & Glick, 2014; Waltimo, 1991; Voros-Balog et al., 2003). Geographic tongue in some patients has been reported to be associated with consumption of special foods such as cheese (Scully, 2004; Korting et al., 1996; Champion et al., 1998). Female hormones may also induce or intensify geographic tongue lesions (Greenburg & Glick, 2014; Waltimo, 1991).

Geographic tongue does not require treatment in most cases (Assimakopoulos et al., 2002). Symptomatic treatment includes mouthwashes containing such anesthetizing compounds as benzydamine, antihistamines like diphenhydramine or corticosteroids like betamethasone (Redman et al., 1972). The present research was carried out to determine the prevalence of geographic tongue and its related predisposing factors in students aged 7-18 in Kermsnhah, Iran in 2014.

## 2. Materials and Methods

This study was approved by the Research Deputy of Kermanshah University of Medical Science, Kermanshah, Iran (#93097). In this descriptive cross-sectional study, data were obtained from the male and female elementary shool, middle school and high school (first and second grade) students in three municipal regions of Kermanshah through observing the students and recording the data collection forms. The following formula was used to determine the sample size, where P is the primary estimate of the given incidence which was considered 1.2 according to a similar study conducted in Kerman, Irab (Hashemipoor et al., 2009) and acceptable error of estimate which was considered 0.012. Therefore, the calculated sample size with confidence level of 95% was 2,817 subjects. A total of 3,600 students were entered into the study.


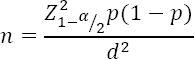


The required data were collected through examination and completing a questionnaire. The responce rate was 98.5%. After taking a history of the patients (including demographic characteristics and medical history), the clinical examinations were performed by general dentists. The tongue was examined using gloves, tongue blade, gauze pad, and flash light. The obtained data were recorded in the questionnaire. All students participated in this study after obtaining the informed consent from their parents. The statistical analyses were performed by the SPSS-20 and the Chi-square test. The P-value less than 0.05 was considered statistically significant.

## 3. Results

In this study, a total of 3,600 students were recruited as the study sample. They were selected from 18 female and male schools (1,800 females and 1,800 males); 1800 elementary students, 900 middle school students and 900 high school students (Table 1). From the total 3,600 students, 283 students (7.86%) suffered from geographic tongue; 122 (43.11%) female students and 161 (56.89%) male students (Table 1).

**Table 1:** Prevalence of geographic tongue in terms of gender and level of education

Variable		Total number	With geographic tongue	Prevalence	p
Gender	Female	1800	122	6.78	0.013
	Male	1800	161	8.94	
Level of education	Elementary school	1800	150	8.33		
	Middle school	900	52	5.78	0.023
	High school	900	81	9.00	
Total		3600	283	7.86	

*chi-square.

From the total of 283 students with geographic tongue, 150 students (53%) were elementary students (7-11 years old), 52 students (18.4%) were middle school students (12-14 years old) and 81 students (28.6%) were high school students (14-18 years old). The results of the Chi-square test indicated that prevalence of geographic tongue was significantly different among three levels of education (p<0.02), so that the prevalence in middle school students was significantly lower than in elementary students (p<0.02) and in high school students (p<0.03). Moreover, the prevalence of geographic tongue in elementary school students was sgnificantly higher than in the high school students (p<0.05) (Table 1).

On the other hand, the prevalence of geographic tongue in female and male students were 43.1% (n=122) and 56.9% (n=161), respectively. Accordingly, the prevalence of geographic tongue in male students was significantly higher than in female students (p<0.01) (Table 1).

Among 283 students with geographic tongue, 132 students (46.7%) had fissured tongue and 68 (24%) had scalloped tongue. The findings of the Chi-square test showed no statistically significant difference between the patients with fissured tongue and those without fissured tongue (p=0.26). However, the proportion of the students with scalloped tongue to those without scalloped tongue was statistically significant in students with geographic tongue (p<0.001), indicating a lower proportion for students with scalloped tongue (Table 2).

**Table 2:** Data concerning the presence of psoriasis, fissured tongue and scalloped tongue in 283 students with geographic tongue

	Number	Percentage	p
Psoriasis	0	0	
Fissured tongue	123	46.64	0.259
Scalloped tongue	68	24.02	<0/001

*chi-square.

On the other hand, in students with geographic tongue, the proportion of the students with pain and discomfort while eating some foods was higher than the students without these symptoms, indicating a statistically significant difference (p<0.02) (Table 3).

**Table 3:** Data concerning the presence of pain and discomfort while eating foods

Variable	Presence	Frequency	Percentage	p
pain and irritation while eating food	Yes	162	57.24	0.015
	No	121	42.76	
	Total	283	100	

*chi-square.

## 4. Discussion

The results of the present study indicated the frequency of 7.86% for geographic tongue in 7-18 year-old students in Kermanshah, which is similar to the findings of the studies carried out in Iran such as the frequency of 7.8% reported by Honarmand (2012), the frequency of 7.6% reported by Rabiei (2006), Taheri and Maleki (2000), and frequency of 6.2% reported by Ghodsi (2004).

On the other hand, the prevalence rates reported in other studies were 12.8%, 12.4%, 1.5%, and 1.8%. The differences between such results are probably due to the difference between the sample size and the population of study.

In the present study, the prevalence of geographic tongue in males was more common than in females. Likewise, Voros-Balog et al (2003) reported the prevalence of geographic tongue to be higher in males than in females (Voros-Balog et al., 2003).

However, prevalence of geographic tongue in both males and females was reported to be similar in the study performed by Kovac-Kovic and Skaleric (2000). In other studies by Taheri and Maleki (2000), Mumcu et al. (2005), and Jainkittivong and Langlaids et al. (2005), this phenomenon was more prevalent in females than in males. This difference in the results of various studies can be due to the age of the study population and interference of hormones. Some studies have reported the severity of these lesions with the start of menstrual cycle, pregnancy and consumption of oral contraceptive pills (OCP) (Greenburg & Glick, 2014; Waltimo, 1991; Neville et al., 2014). In the present study, low occurrence of this phenomenon in girls can be because of their age group, which is more limited in terms of interference of female hormones. Hormones, especially female hormones, may interfere with the induction or intensification of geographic tongue lesions (Greenburg & Glick, 2014; Waltimo, 1991; Neville et al., 2014). Further studies, however, are suggested to analyze and confirm female hormones have such capacity.

The results of this study revealed an increasing feeling of tongue burning and irritation while eating hot and spicy foods as well as eggplant and tomato in 162 patients. In line with the findings of the present study Geographic tongue in some patients have been reported to be associated with consumption of special foods such as cheese (Scully, 2004; Korting et al., 1996; Champion et al., 1998). Voros-Balog et al. (1999) conducted a study on 5034 people and showed that 56% of people with geographic tongue had a history of allergy, which indicated a significant correlation between the two variables.

Furthermore, no significant difference was observed between students with fissured tongue and those without fissured tongue among the students with geographic tongue. Geographic tongue and fissured tongue may be seen simultaneously. It seems that fissured tongue should be considered as the last stage of geographic tongue (Greenburg & Glick, 2014), and since the present study was conducted on the age group of 7-18, fissured tongue is possible to occur in these patients in the future. Honarmand et al. (2012), Voros-Balog et al. (1999) and Ghose et al (1982) identified a significant correlation between geographic tongue and fissured tongue and reported a genetically significant relationship between these two variables in males.

Additionally, the findings of the present study showed no case of psoriasis disease in patients with geographic tongue, which in fact cannot be indicative of presence or lack of relationship between these two diseases. Geographic tongue is considered as a primary sign for the prevalence of psoriasis, and because of the low age of the population in this study, psoriasis is possible to occur in these people in the future. The results of the present study can also be due to mild symptoms of the disease or lack of knowledge of the children about psoriasis disease. Further investigations are recommended to analyze the correlation between psoriasis and geographic tongue.

## 5. Conclusion

The prevalence of geographic tongue in 7-18 year-old students in Kermanshah was 7.86%. The incidence of this lesion was significantly higher in males than in females (p<0.01). There was not any relationship between geographic tongue and psoriasis or fissured tongue in students with this lesion. The proportion of patients with, pain and discomfort while eating some foods was significantly higher than those with no history of these symptoms (p<0.02).

**Recommendations**

Further studies are suggested to analyze the risk factors of this lesion, especially the effects of female hormones. Although this study indicated no relationship between the geographic tongue and fissured tongue or psoriasis, cohort studies are required to prove the existence of such relationship.
